# Aerotolerant capacity of the lung symbiont *Prevotella melaninogenica*

**DOI:** 10.1128/jb.00142-26

**Published:** 2026-07-14

**Authors:** Claire Albright, Gouri Anil, Jacob Evans, Souzane Ntamubano, Ariangela Kozik

**Affiliations:** 1 Department of Molecular, Cellular, and Developmental Biology, University of Michigan 1259, Ann Arbor, Michigan, USA; 2 Department of Computational Medicine and Bioinformatics, University of Michigan 1259, Ann Arbor, Michigan, USA; 3 Division of Pulmonary and Critical Care Medicine, Department of Internal Medicine, University of Michigan 1259, Ann Arbor, Michigan, USA; University of Virginia School of Medicine 12349, Charlottesville, Virginia, USA

**Keywords:** oxidative stress, *Prevotella*, adaptation, microaerobe, tolerance

## Abstract

*Prevotella melaninogenica* is a core member of the human oral and respiratory microbiomes, often representing more than 10% of microbial populations in both healthy and diseased lungs. Despite its prevalence in these oxygenated environments, *P. melaninogenica* has been historically classified as a strict obligate anaerobe, ostensibly unable to survive oxygen concentrations exceeding 0.05%. This creates a fundamental biological paradox as the organism consistently persists in the lower respiratory tract where oxygen levels reach higher than what is tolerated by obligate anaerobes. In this study, we resolve this contradiction by evaluating the growth and tolerance of *P. melaninogenica* across intermediate oxygen concentrations of 2%, 5%, and 8%. Contrary to the long-standing classification, we demonstrate that *P. melaninogenica* maintains growth at 2% and 5% oxygen—a level significantly higher than previously reported for the genus—and exhibits robust aerotolerance in 21% O_2_. Transcriptional profiling via RNA-sequencing reveals that this survival is likely driven by the robust expression of oxidative stress defense and DNA repair machinery. Ultimately, these results provide the first evidence of the specialized mechanisms that enable *Prevotella melaninogenica* to adapt to and colonize the respiratory tract, providing a clearer understanding of its persistence in oxygen-exposed human niches.

**IMPORTANCE:**

This study provides a correction to the 100-year-old classification of *Prevotella melaninogenica* as a strict obligate anaerobe. We demonstrate that this key member of the human microbiome is capable of robust growth under oxygen levels previously thought to be lethal. By identifying transcriptional responses associated with growth and survival, we predict how *Prevotella melaninogenica* dominates the oxygenated niches of the respiratory tract. This work reveals the putative mechanisms driving the adaptive evolution of *Prevotella melaninogenica* and its role in human airway ecology.

## INTRODUCTION

*Prevotella melaninogenica* is classified as a gram-negative bacterium, typically appearing as nonmotile short rods or coccobacilli (1). Since its initial isolation by Oliver and Wherry in 1921, this organism has garnered increasing attention, especially with the development of cultivation-independent approaches that have revealed its significant presence within the human respiratory tract microbiome. Estimates suggest that *P. melaninogenica* comprises around 4%–8% of the oral microbiome (1, 2) and is highly represented in the lung microbiome, accounting for roughly 10% of microbial populations in healthy lungs and up to 13% in individuals with respiratory disease (3). Research has implicated *P. melaninogenica* not only in respiratory health (4) but also in a spectrum of respiratory disorders, including long-term COVID, gastroesophageal reflux disease (5, 6), chronic obstructive pulmonary disease (COPD) (7), and cystic fibrosis (8).

The frequent detection of *P. melaninogenica* in the lungs, where regional microenvironments can range from hypoxic to highly oxygenated (9.9% oxygen (9), contrasts sharply with traditional views that describe this organism as a strict obligate anaerobe, unable to survive in oxygen concentrations exceeding 0.05% (10, 11). This paradox exposes the fundamental gaps in knowledge regarding the organism’s mechanisms for survival and persistence within the lower respiratory tract.

Historically, studies have typically characterized *P. melaninogenica* as strictly anaerobic; however, these conclusions were based on assessments limited to binary comparisons between fully anaerobic and ambient air conditions, without a consideration for intermediate oxygen concentrations (12). Consequently, the bacterium’s potential for growth in intermediate-oxygen environments remains unrecognized. Furthermore, while aerotolerance has been described in two members of the *Prevotella* genus (13), the aerotolerance of *P. melaninogenica* remains unexplored. Within the same order, *Bacteroidales*, related species such as *Bacteroides thetaiotaomicron and Bacteroides fragilis* have demonstrated the ability to proliferate under minimal oxygen levels of 0.14% O_2_(14–16). However, *B. fragilis* has been found to contain an extremely robust aerotolerance, able to tolerate, but not grow, in 21% O_2_ for 48 h (17–19). Furthermore, *B. fragilis* has been found to possess several oxidative stress defense mechanisms (20, 21) similar to those found in facultative anaerobes and is predicted to be equipped with components of the electron transport chain (16, 22–26). To date, no studies have evaluated the growth of *P. melaninogenica* within the intermediate oxygen concentrations characteristic of the lower respiratory tract (1%–9.9% O_2_) (9), coupled with significant aerotolerance to ambient air encountered in the upper respiratory tract. With these considerations in mind, the present investigation aims to address the extent to which *P. melaninogenica* tolerates and grows in oxic environments. Additionally, we seek to elucidate possible oxygen defense strategies employed by this organism, informed by recent findings from related anaerobic bacteria.

## RESULTS

### *P. melaninogenica* can grow in 5% oxygen and survive in 21% oxygen

**Fig 1:**
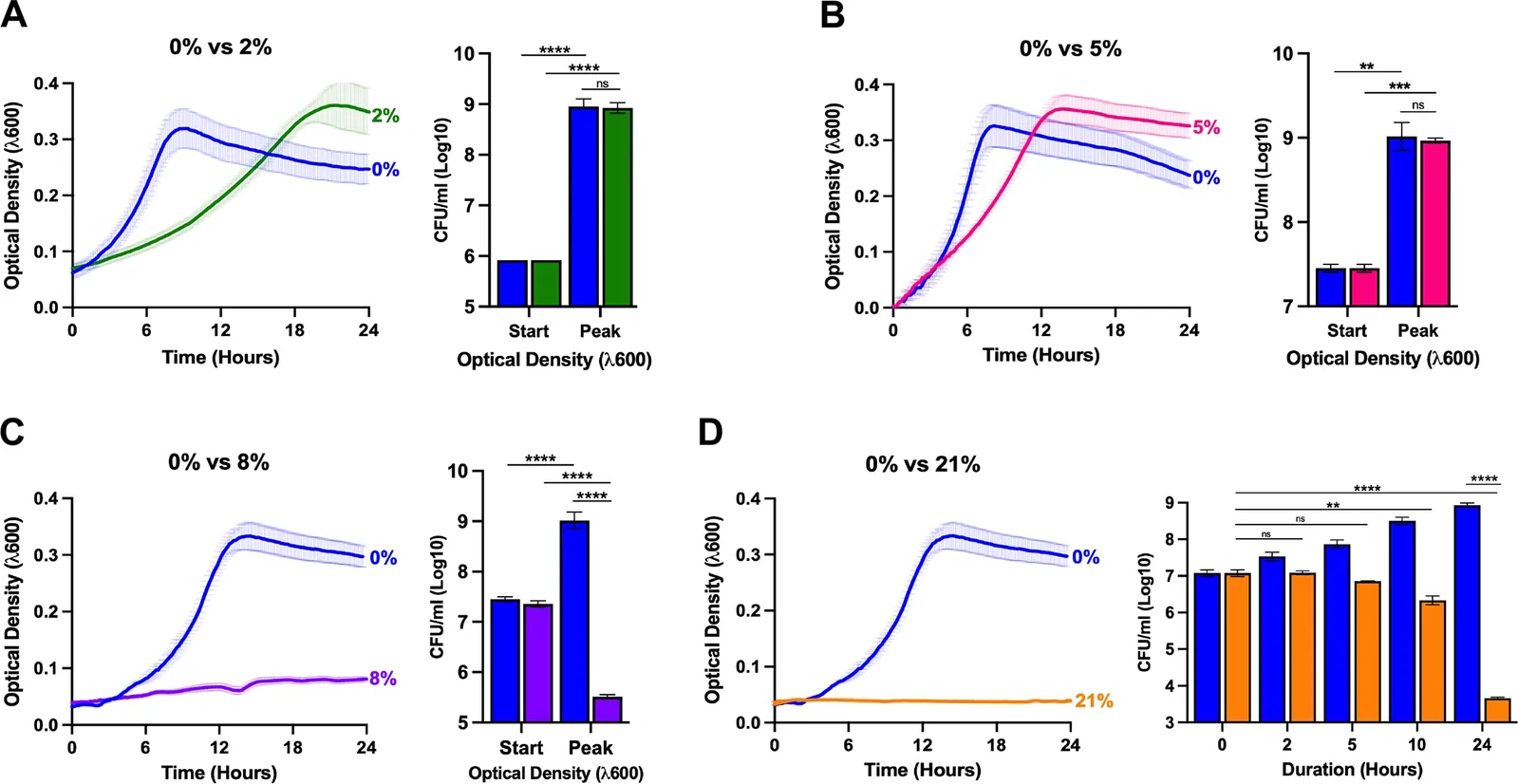
*Prevotella melaninogenica* grows in oxygen and remains tolerant when growth is inhibited at higher oxygen concentrations. (**A**) *P. melaninogenica* simultaneously grown in 0% O_2_ (blue) and 2% O_2_ (green) over 24 h. Optical density was measured at the wavelength 600. CFU at the culture’s starting OD (start) vs at the culture’s respective peak OD (peak) is shown for 0% (blue) and 2% (green) cultures with a bar graph. (**B and C**) Similar to A, growth curves and CFU plots are shown for 0% vs 5% O_2_ and 0% vs 8% O_2_. (**D**) Similar to panels **A–C**, parallel growth curves in 0% vs 21% are shown. However, instead of taking CFU based on the OD, the CFU from 0% and 21% was plated at defined time points (0, 2, 5, 10, and 24 h) to demonstrate the death curve in ambient air.

Our objective was to determine the growth dynamics and aerotolerance profile of *P. melaninogenica* in low-oxygen conditions. To do this, cultures were exposed to a range of oxygen conditions: 2%, 5%, 8%, and 21% oxygen. We first tested the 2% O_2_ condition against the anaerobic control (Fig. 1A). Both cultures had identical inoculums, as indicated by the culture colony-forming unit (CFU) at the 0 h. The replication rate in 2% O_2_ was about half as fast as in the 0% control; however, there was no significant difference in the peak optical density or CFU (plated at the peak OD) of the two cultures. Next, to assess the impact of a moderate increase in oxygen, we tested 5% O_2_ vs 0% O_2_ (Fig. 1B). Similar to 2%, the replication rate in 5% O_2_ was about half as fast as in the 0% culture run in parallel. Similar to the 2% results, there was no significant difference in the maximum optical density or CFU at the peak OD. We then tested 8% O_2_ vs 0% O_2_ (Fig. 1C). *P. melaninogenica* cultures did not grow in 8% O_2_ over the course of 24 h, indicating that the upper threshold for growth falls between 5% and 8% O_2_. The culture CFU at 0 h was ~10^7.3^, confirming initial cell viability. After 24 h, the culture CFU significantly decreased to ~10^5.5^, but was not reduced to 0. Having found the upper limit for growth, we next aimed to test the aerotolerance of *P. melaninogenica* by testing at what point the cells begin to lose viability in fully aerobic conditions, (i.e., 21% O_2_) compared to the 0% O_2_ control (Fig. 1D). As expected, *P. melaninogenica* cultures did not grow in 21% O_2_ over the course of 24 h. The culture CFU at 0 h was ~10^7^, demonstrating cell viability at the time of inoculation. Strikingly, there was no significant decrease in culture CFU by 2 and 5 h. By 10 h, there was a slight decrease in CFU to ~10^6^. By 24 h, the culture CFU had significantly decreased to ~10^3.5^, but surprisingly was not eliminated. From these data, we successfully determined the upper limit of *P. melaninogenica* growth to be between 5% and 8%, with no significant difference in maximum OD or cell viability at 5% compared to 0% O_2_. Second, the sustained presence of viable cells after 24 h at 8% and 21% O_2_ demonstrates significant aerotolerance even in aerobic conditions.

### *P. melaninogenica* consumes oxygen

**Fig 2:**
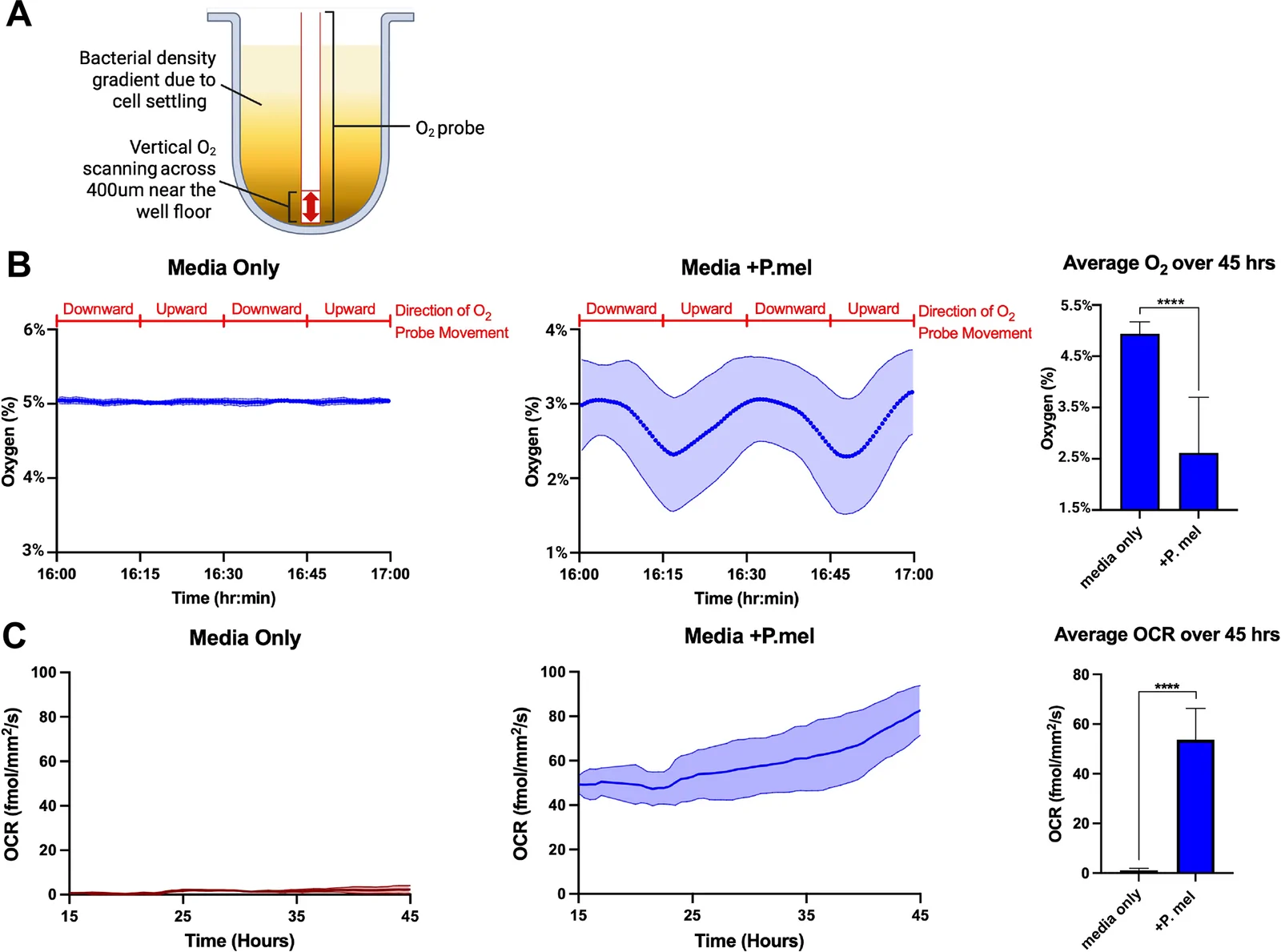
*Prevotella melaninogenica* consumes oxygen. (**A**) Model of the Resipher oxygen probe used. A well with 100 μL bacterial culture and the loaded O₂ probe is pictured. The area of vertical scanning is represented with a red arrow. (**B**) Oxygen measurements as the probe moves up and down across 400 μm over the span of 1 representative h (16 h was chosen) in an atmosphere of 5% O₂. The Y-axis is percent oxygen in the culture. X-axis is time (h:min). The red line above graphs represents the probe depth over the course of the hour. Left graph: O₂ of BHI medium-only cells. Right graph: O₂ of BHI media + *P. melaninogenica*. Bar graph depicts the average oxygen level of BHI medium-only cells and BHI media + *P. melaninogenica* over 45 h. (**C**) Oxygen consumption rate (OCR) quantification over time in an atmosphere of 5% O₂. The Y-axis is the OCR (femtomole/square millimeter/seconds). The X-axis is time (h). Left graph: OCR of BHI medium-only cells. Right graph: OCR of BHI media + *P. melaninogenica*. The bar graph depicts the average OCR level of BHI medium-only cells and BHI media + *P. melaninogenica* over 45 h.

Our next objective was to determine if *P. melaninogenica* consumes oxygen. This is necessary to better understand how *P. melaninogenica* is influencing its environment in the respiratory tract. Our approach was to determine the effect the presence of *P. melaninogenica* has on the surrounding oxygen concentration in liquid media. To do this, we measured the O_2_ concentration and the cellular oxygen consumption rate (OCR) within the liquid cultures, with a medium-only control to confirm full diffusion of O_2_ into the media and heat-killed control to confirm the absence of OCR without live cells (Fig. 2B and C; Fig. S1). When examining the O_2_ concentration in the liquid cultures with live *P. melaninogenica*, we find that the percent O_2_ varies significantly across well depths (Fig. 2B). This finding suggests that a lower oxygen level is correlated with a higher cell density as cell settling leads to higher cell density at the bottom of each well. When looking specifically at the representative 16–17 h time point, we see that at the highest point in the probe’s 400 μm range (16 h:00 min and 16 h:30 min), the O_2_ concentration is ~3%. However, at the bottom of the 400 μm range (16 h:15 min and 16 h:45 min), the O_2_ concentration is ~2.4%, showing a striking 0.6% change in O_2_ over the span of 400 μm (Fig. 2B). In contrast, the medium-only control demonstrates no change in its 5% O_2_ concentration throughout the probe’s vertical scanning over the 16–17 h time point (Fig. 2B). When examining the average O_2_ concentration of the medium-only control vs the media + *P. melaninogenica* culture over the entire 45-h run, the overall change in the oxygen level becomes extremely apparent, with the medium-only control demonstrating 5% O_2_ and the +*P. melaninogenica* culture demonstrating an average of 2.5% O_2_. Therefore, we found that the O_2_ decreases by ~2.5% in the presence of *P. melaninogenica* (Fig. 2B). When examining the cellular oxygen consumption rate (OCR) in the liquid brain heart infusion (BHI) cultures with *P. melaninogenica*, we find that the OCR is ~50 (fmol/mm^2^/s) at 15 h and that the OCR increases to ~80 (fmol/mm^2^/s) by 45 h (Fig. 2C). By contrast, the medium-only negative control shows no significant change in OCR from 0 (Fig. 2C). When examining the average OCR of the medium-only control vs the media + *P. melaninogenica* culture over the entire 45-h run, we again see that the OCR of the negative control is 0 and the OCR of the *P. melaninogenica* culture is ~50 (fmol/mm^2^/s). When testing heat-killed cells, we find no OCR present (Fig. S1). Based on these results, we determined that *P. melaninogenica* has a significant impact on its surrounding environment through the consumption of oxygen.

Given these striking observations, we then surveyed the genome to identify candidate genes that may be associated with oxidative stress defense in *P. melaninogenica,* based on the defense systems that have been worked out in the study of *E. coli*. After the identification of *E. coli* oxidative stress genes(27), we then identified whether those genes were predicted to be present in the *P. melaninogenica* ATCC 25845 genome. This analysis resulted in the identification of 16 genes. All genes are protein-coding; therefore, along with the ENSEMBL gene IDs, the protein symbols and names are listed. Therefore, we developed a list of 16 genes predicted to be involved in the oxidative stress defense system in *P. melaninogenica* (Table 1).

**TABLE 1:** Putative oxidative stress defense genes in *P. melaninogenica*. Gene symbol, protein symbol, protein name, and ENSEMBL gene IDs are listed

Gene symbol	Protein symbol	Protein name	Gene ID
*nqrA*	NqrA	Na(+)-translocating NADH-quinone reductase subunit A	ENSB:BLK3DzihWAUCU2h
*nqrB*	NqrB	Na(+)-translocating NADH-quinone reductase subunit B	ENSB:0ts2B8ǪxmfumI8k
*nqrC*	NqrC	Na(+)-translocating NADH-quinone reductase subunit C	ENSB:ozXIVafGGc-_t5w
*nqrD*	NqrD	Na(+)-translocating NADH-quinone reductase subunit D	ENSB:WkcJKkkqZISfMTL
*nqrE*	NqrE	Na(+)-translocating NADH-quinone reductase subunit E	ENSB:ywTzU-23lǪPODR6
*nqrF*	NqrF	Na(+)-translocating NADH-quinone reductase subunit F	ENSB:ZW47XrVedhrYBqF
*appC*	CydA	High affinity Cytochrome bd-I ubiquinol oxidase subunit A	ENSB:hgrg4FXl3GU5Tj7
*cydB*	CydB	High affinity Cytochrome bd-I ubiquinol oxidase subunit B	ENSB:1etJFLlraSMBMaO
*ahpF*	AhpF	Alkyl hydroperoxide reductase F	ENSB:6tkqKtObKdEP_dG
*ahpC*	AhpC	Alkyl hydroperoxide reductase C	ENSB:5lcptuqVuosM1zm
*rbr*	Rbr	Rubrerythrin	ENSB:1Puk-3YEHTBYvYC
*bcp*	Bcp	Bacterioferritin-comigratory protein	ENSB:YǪDZ8uEFXǪbgFJh
*dfx*	Dfx	Desulfoferrodoxin	ENSB:t69hwx3lFW54Tu1
*ftnA*	FtnA	Bacterial non-heme ferritin	ENSB:oGWjtJqVADOcRC4
*rd*	Rd	Rubredoxin	ENSB:7gVWY0rTVZkYqec
*oxyR*	OxyR	Hydrogen peroxide-inducible gene activator	ENSB:Ǫy6VxrVcvIefsnp

### Sixteen putative oxidative stress defense genes were significantly upregulated in 2% oxygen

**Fig 3:**
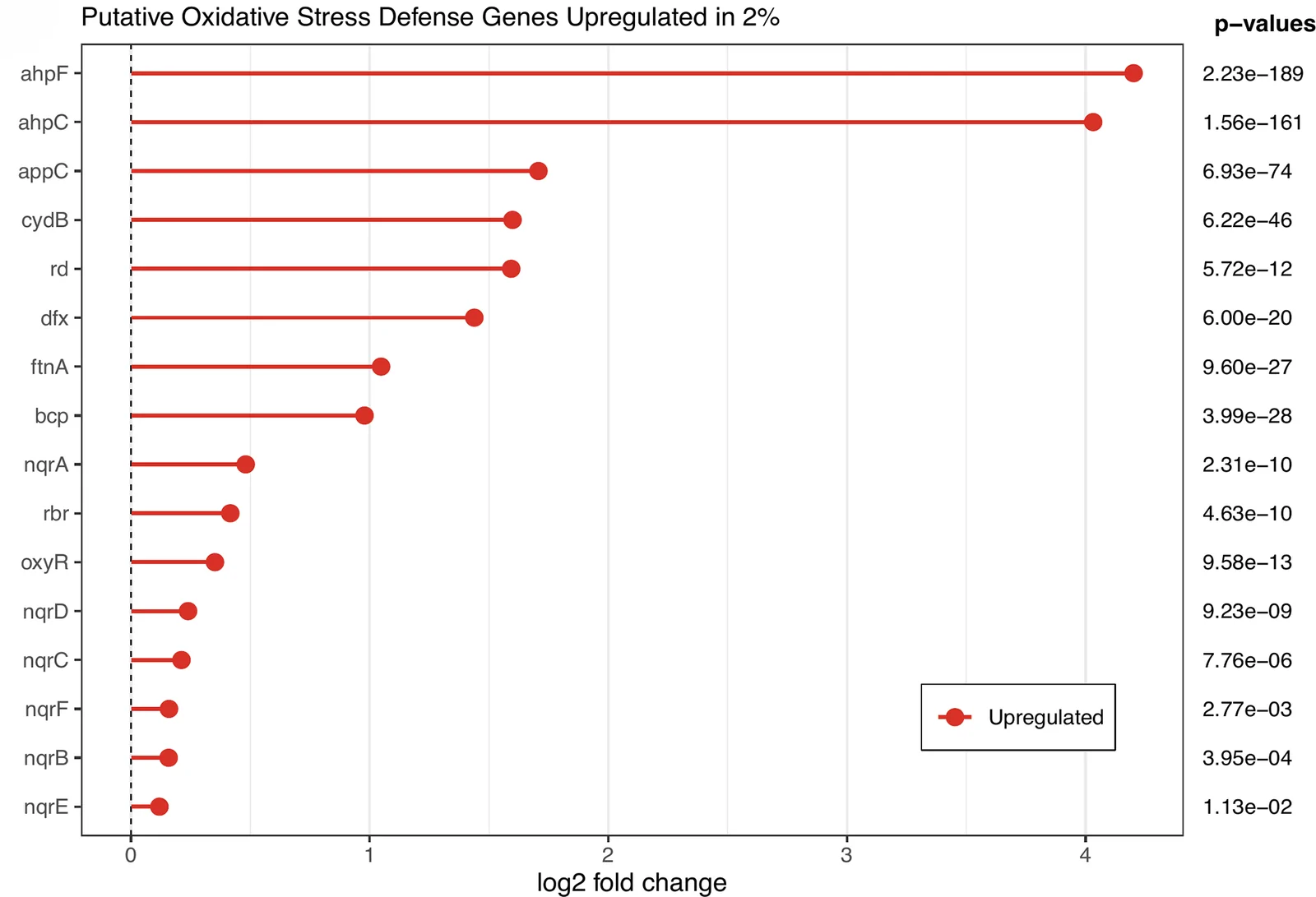
All putative oxidative stress defense genes are significantly upregulated in 2% oxygen vs 0% oxygen. The y-axis is labeled with gene symbols. The x-axis is log 2 fold change from gene expression in 0% O_2_. Adjusted *P* values of each respective gene are listed.

Our next objective was to determine if the genes predicted to be associated with oxidative stress defense are significantly upregulated under low-oxygen conditions compared to anaerobic conditions. We performed total RNA sequencing to quantify the transcriptomic profile of *P. melaninogenica* grown within 2% vs 0% O_2_ environments. Analysis focusing specifically on our 16 genes of interest revealed that *all* candidates were significantly upregulated in the 2% O_2_ condition compared to their respective anaerobic (0% O_2_) expression levels (Fig. 3). Genes *ahpF and ahpC* were the two most significantly upregulated genes by a substantial margin (Fig. 3 and Table S1). In addition, many of the 16 genes have basal transcript counts over the median and average of all 2,307 genes predicted in *P. melaninogenica’s* genome. Thus, even a modest fold change may represent a substantial shift in the absolute mRNA pool and likely has biological meaning (Table S3). Further analysis with STRING predicted significant interactions between the proteins encoded by these 16 genes (Fig. S2). These results demonstrate that the genes we predicted to be associated with oxygen defense and tolerance are in fact significantly upregulated in the low-oxygen 2% condition.

### Putative DNA protection and repair genes were significantly upregulated in 2%

**Fig 4:**
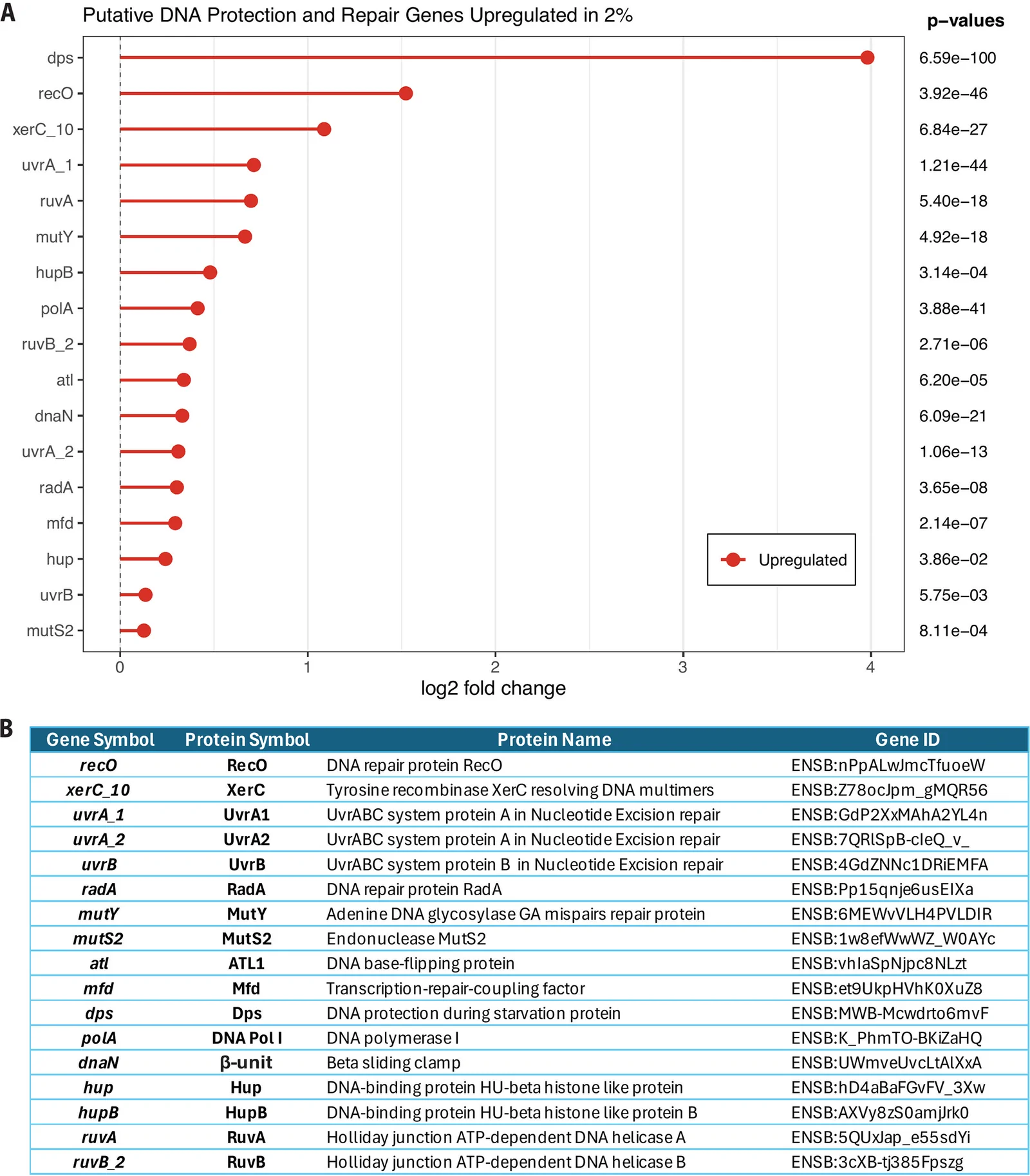
Putative DNA integrity and repair genes are significantly upregulated in 2% oxygen vs 0% oxygen. (**A**) Upregulated genes with gene labels on the Y-axis and log 2 fold change on the X-axis. Adjusted *P* values of each respective gene are listed. (**B**) Putative integrity and repair gene table based on the transcriptomic results. Gene symbol, protein symbol, protein name, and ENSEMBL ID are shown.

Our next objective was to identify if there were any groups of significantly upregulated genes with functions outside oxidative stress defense. In the transcriptomic data set described above, we found 17 genes associated with DNA protection and repair in *E. coli* were significantly upregulated in *P. melaninogenica* growing in 2% O_2_ when compared to the basal anaerobic expression levels (Fig. 4A). The log2 fold change of each upregulated gene is listed in Table S2. Additional information for each of the 17 genes is shown in a table (Fig. 4B). All 17 genes are protein-coding; therefore, the protein symbols and names are shown in addition to the gene ENSEMBL IDs. These results demonstrate that there are many genes associated with DNA protection and repair that are significantly upregulated in 2% oxygen.

### Putative oxidative stress defense system homology is found across the *Prevotella* genus

**Fig 5:**
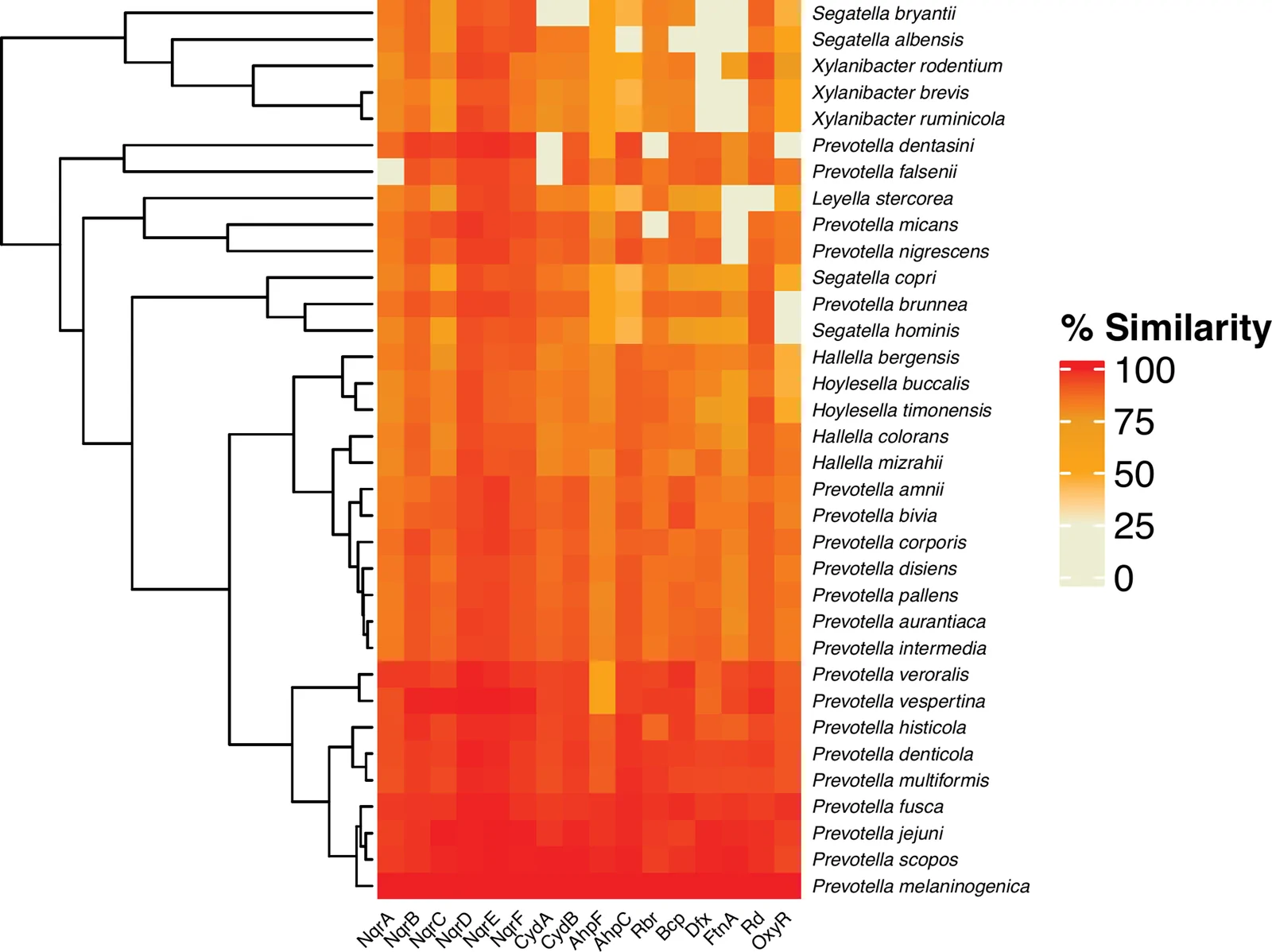
Homologs of the identified putative oxidative stress defense proteins upregulated in *P. melaninogenica* are nearly ubiquitous across the *Prevotella* genus but less prevalent in the reclassified *Prevotella* species. The y axis denotes the 21 *Prevotella* species (primarily associated with the respiratory tract) and 13 reclassified *Prevotella* species (associated with the gut and vagina). The x-axis denotes proteins encoded by the 16 upregulated putative oxidative stress defense genes in *P. melaninogenica*. The top BLASTp hit with >60% coverage and <0.05 E value within each bacteria for each protein is shown. The percent amino acid similarity of each candidate homolog is depicted with a color gradient (red = 100%; cream = 0%). Bacteria are clustered based on protein homology.

Finally, we sought to identify protein homologs across the *Prevotella* genus and formerly classified *Prevotella* species to the identified putative oxidative stress defense proteins in *P. melaninogenica*. Due to the association of the *Prevotella* genus within the oxygenated respiratory tract and the association of reclassified *Prevotella* species within the vagina (hypoxic) and gut (anoxic), we sought to examine the potential association of oxidative stress defense machinery and habitat oxygen levels. To determine the homology, we looked for amino acid sequence similarity to the putative oxidative stress defense proteins from *P. melaninogenica* across the genomes of the 21 *Prevotella* species and the 13 reclassified species commonly found in the gut and vagina. Across all 21 *Prevotella* species, 11 of the 16 analyzed proteins demonstrated amino acid similarity to those in *P. melaninogenica*: NqrB, NqrC, NqrD, NqrE, NqrF, CydB, AhF, AhpC, Bcp, Dfx, and Rd (Fig. 5). The average amino acid similarity for each protein across the genus was between 81% and 97%. However, several proteins exhibited lineage-specific absence. Specifically, NqrA, CydA, Rbr, FtnA, and OxyR homologs, which otherwise shared high amino acid similarity across the remaining species, showed a complete absence (0% similarity) in distinct subsets of *P. falsenii*, *P. dentasini*, *P. micans*, *P. nigrescens*, and *P. brunnea*. Hierarchical clustering of *Prevotella* and reclassified *Prevotella* species based on protein similarity heatmaps revealed six distinct groupings (Fig. 5). These groupings demonstrate a progressive increase in amino acid similarity relative to *P. melaninogenica*, with a 9-species core group sharing a high mean amino acid similarity of 96.3% with *P. melaninogenica*. From these data, we successfully determined protein homology to the identified putative oxidative stress defense proteins in *P. melaninogenica* across the *Prevotella* genus and formerly classified *Prevotella* species.

## DISCUSSION

This study demonstrates that *Prevotella melaninogenica*, typically classified as a strict obligate anaerobe, possesses the capacity for robust growth and survival in oxygenated environments. Our results indicate that while the upper limit for active growth is between 5% and 8% oxygen, the organism exhibits significant aerotolerance even at atmospheric levels (21% O_2_), maintaining viable subpopulations for at least 24 h. We propose that this survival is underpinned by active modification of its local environment through oxygen consumption and a sophisticated, oxygen-induced transcriptional response. Specifically, we observed significant upregulation of 16 putative oxidative stress defense genes, such as the Na^+^-NQR (*nqrABCDEF*), the high-affinity cytochrome *bd I* oxidase (*cydAB*), and the superoxide reductase (SOR) desulfoferrodoxin (*dfx*), alongside 17 genes dedicated to DNA protection and repair, most notably “DNA protection during starvation” (*dps*) (28). Furthermore, protein homology analysis reveals that these oxidative defense mechanisms are highly conserved across the *Prevotella* genus, particularly among species inhabiting the human respiratory tract. Collectively, these findings provide a necessary correction to the century-old classification of *P. melaninogenica* and provide evidence that its persistence in the oxygenated human airway is likely driven by a specialized molecular toolkit for oxygen defense and DNA maintenance.

Our data demonstrate that *P. melaninogenica* proliferates robustly in 2% and 5% oxygen, achieving a maximum optical density and cell viability comparable to anaerobic controls, with only a marginal reduction in the replication rate. This discovery challenges a century of classification, beginning with Oliver and Wherry (1921), who designated the organism as an obligate anaerobe based primarily on its inability to grow in ambient air. The classification of strict anaerobe has been upheld by a reliance on binary experimental frameworks, comparing only 0% to 21% O_2_, while leaving intermediate concentrations largely unexplored (12). Early attempts to examine modular oxygen levels, such as those by Rosenblatt (1975) and Loesche (1969), were hindered by unmeasured oxygen concentrations, qualitative metrics, or the use of nonstandardized clinical strains, making their results unreliable. Furthermore, previous methodologies often focused on transient oxygen exposure or colony morphology on solid media, which does not capture the comprehensive growth kinetics provided by our simultaneous and continuous liquid culture monitoring. Therefore, this study establishes for the first time that *P. melaninogenica* growth at 2% and 5% oxygen is sustained and robust from the point of inoculation. These findings suggest that the physiological growth threshold of this species is significantly higher than previously recognized, necessitating a revision of its classification as a strict anaerobe.

Our results further demonstrate that even when active proliferation is arrested at elevated oxygen concentrations, *P. melaninogenica* maintains sustained aerotolerance for at least 24 h. Notably, in ambient air (21% O_2_), no significant decrease in viability was observed during the first 10 h of exposure, with a persistent subpopulation surviving through the full 24-h period. Our data are supported by the physiology of the closely related *Bacteroides fragilis*, which—despite a lower growth threshold (0%–0.14% O_2_) (14)—exhibits a comparable capacity to withstand 24–72 h of ambient air (21% O_2_) (17, 18). Interestingly, in *B. fragilis*, growth arrest in high-oxygen environments is a regulated process involving the *oxe* gene, suggesting cessation of division may be a strategic entry into dormancy to prioritize survival, rather than a failure of oxidative stress defense. In our study, after 24 h exposure of liquid cultures to 21% O_2_, anaerobic rescue colonies were significantly smaller than those plated from anaerobic control cultures. This observation supports our hypothesis that *P. melaninogenica* enters cell dormancy as an adaptive strategy as it would likely take a longer time to exit this state and restart replication after returning to an environment conducive for growth. In the context of the human lung where oxygen tension can vary dramatically from approximately 9.9% O_2_ in healthy airways to as low as 1% within the thickened mucus of cystic fibrosis patients, this physiological flexibility is critical (9). The capacity of *P. melaninogenica* to grow robustly at 5% while remaining resilient at 21% establishes it as an organism highly adapted to the spatial and physiological heterogeneity of the respiratory tract.

Beyond growth and tolerance, our results demonstrate that *Prevotella melaninogenica* actively modifies its local environment through significant oxygen consumption, with a rate of between 50 and 80 fmol O_2_/mm²/s in 2% O_2_. As oxygen consumption remains uncharacterized in *Prevotella* species, these findings address a significant literature gap regarding the metabolic capabilities of these commensals. While oxygen consumption in *Bacteroides fragilis* reaches 50 nmol/min (9 nmol/min/mg dry cells) in 0.1% O_2_ (14), our findings extend this metabolic trait to *P. melaninogenica* under higher oxidative pressures. This consumption likely serves as an evolutionary adaptation for colonizing niches where oxygen concentrations would otherwise overwhelm oxidative stress defenses. In the context of the highly variable oxygen gradients of the human lungs, this strategy would provide a distinct advantage, likely explaining *P. melaninogenica*’s predominance in the lower airway. Furthermore, by modulating local oxygen levels, *P. melaninogenica* may establish low-oxygen microenvironments essential for the survival of frequently associated taxa, like *Veillonella* and *Fusobacterium* spp. Our data provide novel insights into *P. melaninogenica*’s scavenging capabilities at 2% O_2_, identifying a mechanism of environmental niche modification. These findings suggest *P. melaninogenica* may act as a keystone species facilitating polymicrobial community survival through local redox modulation.

**Fig 6:**
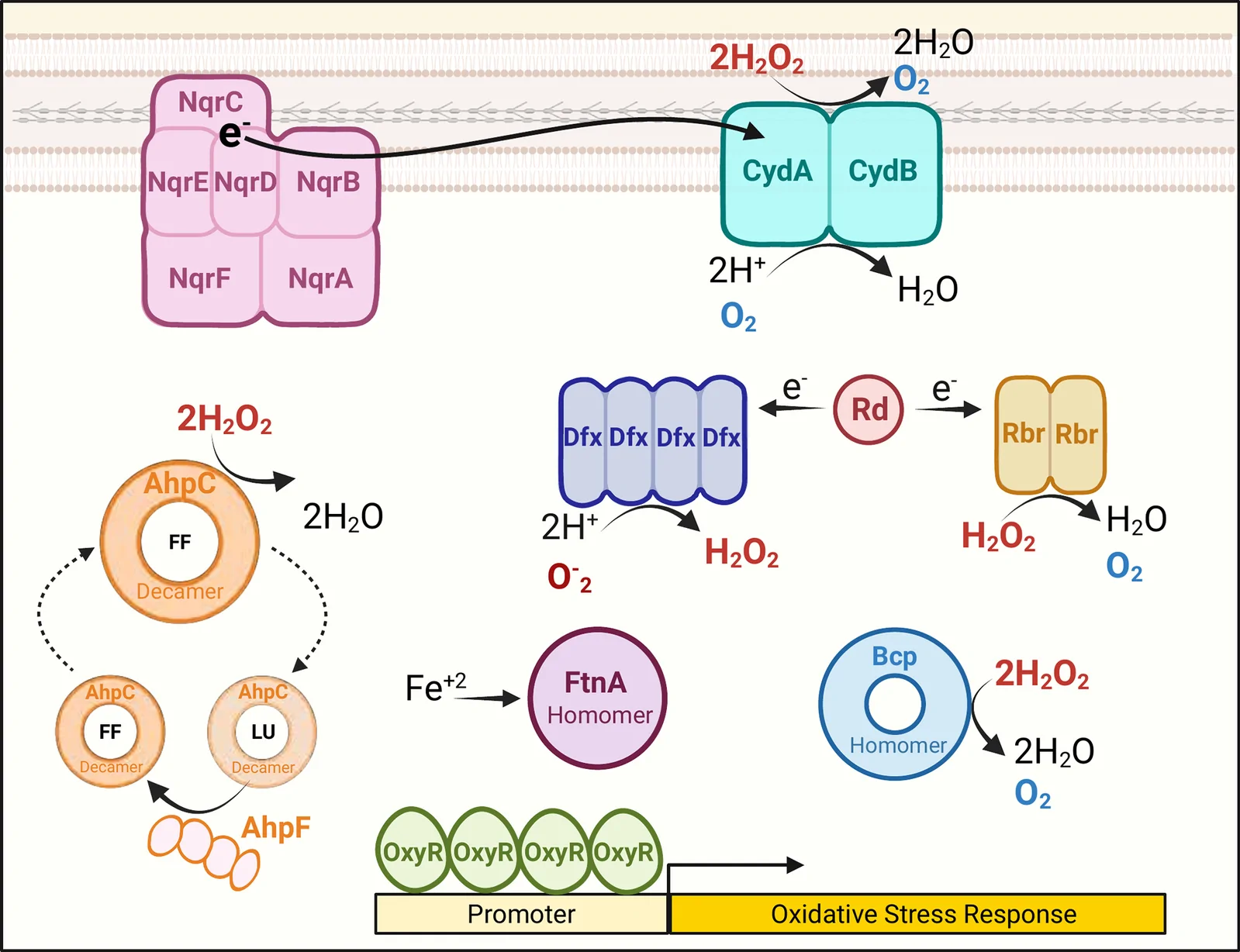
The putative oxygen defense mechanisms upregulated in 2% oxygen to confer tolerance. The membrane-associated Na+-NQR complex is shown in pink, with the six subunits NqrABCDEF labeled in dark pink. The membrane-associated high-affinity cytochrome bd-I (Cyd bd I) is shown in aqua, with its two subunits CydAB labeled in dark aqua. Rubredoxin (Rd) is a red circle, depicting a monomer. Desulfoferrodoxin (Dfx) is a dark blue tetramer complex. Rubrerythrin (Rbr) is a yellow dimer. AhpC is in orange, representing a ring-like decamer. AhpC’s two active site conformations, (fully folded and locally folded) are represented with “FF” and “LU.” AhpF is in orange, representing a homodimer. Bacterioferritin-comigatory protein (Bcp) is a blue ring-like homomer (a complex with many identical subunits). Bacterial non-heme ferritin (FtnA) is a maroon circle that represents another homomer. Finally, the active OxyR tetramer is shown in green on the promoter of the oxidative stress gene operon. Electron flow is depicted with black arrows. Reactive oxygen species (ROS) are depicted in red. Oxygen is depicted in blue.

Our results show that two protein complexes associated with aerobic respiration in low-oxygen environments, NADH:quinone oxidoreductase (Na^+^-NQR) and the high-affinity terminal oxidase Cytochrome bd-I CydAB (29), are significantly upregulated in *P. melaninogenica* under 2% O_2_. Historically, members of the *Prevotella* genus were considered limited to fermentation and anaerobic respiration due to their classification as anaerobes (24, 30). However, genomic sequencing has revealed that several supposedly anaerobic bacteria, such as *B. fragilis*, possess oxygen-directed respiratory systems (25). These low-oxygen respiratory systems comprise an NADH dehydrogenase (Na^+^-NQR and/or NADH2), a quinol electron carrier, and a high-affinity terminal oxidase (CydAB) (31), which catalyze the reduction of oxygen to water. In contrast, anaerobic respiration utilizes fumarate reductase as the terminal oxidase to catalyze the reduction of fumarate to succinate in the absence of oxygen. In *P. melaninogenica*, the genome is predicted to encode Na^+^-NQR, NADH2, menaquinone, fumarate reductase, CydAB, and ATP synthase. Our transcriptomic data show that NADH2 and fumarate reductase are transcribed under anaerobic conditions, Na^+^-NQR and CydAB are significantly more expressed at 2% oxygen, and menaquinone is expressed under both anaerobic and aerobic conditions (Fig. 6). These findings align with the expression patterns observed in *B. fragilis*. Therefore, we propose that under 2% O_2_, *P. melaninogenica* energetically benefits from low levels of oxygen through a type of aerobic respiration.

In addition to energetic concerns, microbes growing in oxygen must also have a strategy to mediate oxidative stress. Our genomic and transcriptomic analysis of *P. melaninogenica* also shows that superoxide dismutase (SOD) is not predicted to be present, yet a superoxide reductase (SOR) identified as desulfoferrodoxin (*dfx*) is present and significantly upregulated in 2% O_2_. SOD is a well-characterized superoxide scavenger in many bacteria, including *B. fragilis* and *E. coli* (32–34). The absence of SOD in the *P. melaninogenica* genome is significant given the enzyme’s importance in ROS detoxification. However, an alternative superoxide scavenger, SOR (previously identified in *Desulfoarculus baarsii* [35], and *Desulfovibrio vulgaris* [36]) is present in *P. melaninogenica* and upregulated under 2% O_2_. This finding is particularly notable as SORs have not been identified in the closely related *Bacteroides* genus. Further genomic analysis of the SOR *dfx* reveals that it is predicted to be present in other *Prevotella* species such as *P. histicola* and *P. intermedia*, suggesting this may represent a distinct feature of the *Prevotella* genus. SOD and SOR are redox-active metalloenzymes that have evolved independently and utilize different catalytic mechanisms. Our data showing the significant upregulation of SOR under aerobic conditions support its role in superoxide detoxification (Fig. 6). These results suggest that *P. melaninogenica* may utilize unique elements within its ROS detoxification machinery that distinguish it (and its oxygen tolerance) from the related *Bacteroides* genus.

Beyond its role in energy generation, cytochrome bd-I (CydAB) has been proposed to enable various essential physiological functions. CydAB was found to have peroxidase activity and enhance stress tolerance in *E. coli* (37, 38), and most notably, drive oxygen consumption in *B. fragilis* (14). Given the robust oxygen consumption by *P. melaninogenica* observed in our study, the need for experimental validation is ground for future study. Consequently, we propose that the significant upregulation of CydAB in *P. melaninogenica* is likely driven by both an energetic benefit and a potential contribution to oxidative stress defense (39).

Our results show that *ahpC* and *ahpF* are the most significantly upregulated genes in *P. melaninogenica* under 2% oxygen, with differential expression levels markedly higher than those of any other significantly upregulated genes. These genes encode peroxiredoxins (Prxs), a peroxidase family essential for antioxidant protection in diverse bacteria (40). In *B. thetaiotaomicron* and *B. fragilis*, *ahpCF* is regulated by *oxyR* and is highly upregulated under oxidative stress, supporting our transcriptomic analysis findings (41–43). Mechanistically, the proteins AhpC and AhpF function as a synergetic unit to catalyze the NADH-dependent reduction of H_2_O_2_ to H_2_O in *E. coli* (40). AhpC serves as the peroxidase that directly reduces H_2_O_2_, while AhpF acts as the reductase required to maintain AhpC in its active, fully folded conformation (40). The exceptional fold-change observed in our data, combined with the established ROS-scavenging roles of these enzymes in *E. coli*, indicates that this conserved system is a critical functional priority for *P. melaninogenica*. These results suggest that AhpC/F protein activity is central to maintaining redox homeostasis within the oxygen-variable environment of the human respiratory tract (Fig. 6).

In addition to putative oxidative stress machinery, we identified many putative DNA repair genes upregulated in 2% O_2_, indicating a previously unknown damage repair response in addition to O_2_ defense in *P. melaninogenica*. DNA repair is thought to be an essential component of the oxidative stress response for any growth in oxygen (44). The DNA repair genes upregulated in *P. melaninogenica* are predicted to fall into the following functional groups: excision repair, replication and recombination processing, mismatch repair, SOS response, and DNA condensation. In *B. fragilis*, DNA repair is regulated by the *recA-*mediated SOS response (45). However, *recA* was not found to be upregulated in *P. melaninogenica* under 2% oxygen. No other proteins previously characterized as DNA repair regulators were found to be upregulated in *P. melaninogenica*; however, the genome of *P. melaninogenica* is not well characterized, resulting in many genes remaining unannotated. It is likely that the regulator of DNA repair in *P. melaninogenica* is a novel protein in need of characterization. We will investigate this in future work.

Finally, given the evidence for robust oxidative defense machinery identified in our data, we investigated whether this repertoire was unique to *P. melaninogenica* or a conserved trait within the newly reorganized genus. Due to the high degree of interspecies diversity, many bacteria formerly within *Prevotella* were recently reclassified (46). The refined *Prevotella* genus now comprises 21 species primarily localized to the human respiratory tract, whereas many reclassified taxa occupy niches with lower oxygen availability, such as the gut and vaginal microbiomes. This suggests a correlation between environmental oxygen levels and the specific repertoire of oxidative defense machinery in the associated microbes. Furthermore, the presence of protein homologs with high amino acid similarity across the genus indicates that the capacity for oxygen growth and tolerance is likely a shared trait rather than one unique to *P. melaninogenica*. These results necessitate a re-evaluation of the physiological classification of the *Prevotella* genus as a whole. This study represents a critical first step in elucidating the adaptive physiology, function, and microbial dynamics of *Prevotella melaninogenica* within the oxygen-variable landscape of the human respiratory tract.

## MATERIALS AND METHODS

### *P. melaninogenica* reconstitution and culture conditions

*Prevotella melaninogenica* 25845 was purchased from ATCC as a frozen culture. Bacteria was grown in Brain Heart Infusion (BHI) (Anaerobe Systems) broth under anaerobic conditions in an AS-500 anaerobic chamber (Anaerobe Systems). Gram stain and Sanger sequencing were performed to confirm the ID.

### *P. melaninogenica* growth and viability assay

For each respective run, the inoculating culture of *P. melaninogenica* was initially grown in Brain Heart Infusion (BHI) broth at 37°C under strictly anaerobic conditions (0% O_2_). All inoculums derived from this culture were then cultured in BHI broth at 37°C in either the anaerobic chamber for 0% O_2_, the hypoxic chamber (Coy) for 2%, 5%, and 8% O_2,_ respectively, or ambient air for 21% O_2_. Upon detection of *P. melaninogenica* growth in 2% O_2_ and 5% O_2,_ respectively, the assays were repeated with the addition of parallel 0% O_2_ cultures inoculated from the same parent culture for direct comparison of the growth rate, maximum optical density (OD), and cell viability over time in 0% vs 2% O_2_ and 0% vs 5% O_2_. Growth kinetics were quantified by measuring optical density (OD) over time at wavelength 600 using AltoMicroplate Readers (Cerillo). For parallel anaerobic and aerobic assays, identical 96-well plates were read by a microplate reader in the anaerobic chamber and hypoxic chamber (at either 2% or 5% O₂), respectively. Cell viability was quantified via colony-forming units (CFU) following anaerobic plating. All cultures were plated at 0 h to establish baseline inoculum viability. For each respective run, the culture was plated anaerobically at 0 h to quantify the number of viable cells in the inoculum via colony-forming units (CFUs). Subsequent anaerobic plating was performed at the time of maximum OD or, in the absence of observed growth, at 24 h. For cultures exposed to 21% O_2_, a death curve was established by plating at 0, 2, 5, 10, and 24 h. Bacterial identity was confirmed using Gram stain and PCR.

### Quantifying significance

An unpaired t test with Welch’s correction was performed to determine *P* values. *P* values of < 0.05 were considered significant. The *P* value range is indicated with 1–4 stars.

### Measuring environmental oxygen and cellular oxygen consumption rate

The Resipher system (Lucid Scientific) was used to quantify the soluble oxygen and cellular oxygen consumption rate (OCR) over time and at varying well depths. The Resipher system consists of a 96-well plate containing inoculums, a sensing lid with probes, the processing *device*, and the base station *hub*. Each probe is coated in a material which, when optically excited, produces fluorescence and light directly proportional to the level of oxygen in solution. In this way, the O_2_ concentration is quantified. The OCR is calculated based on the fmol O_2_ /mm^2^/second. Measurements are taken every 30 s while the probe moves up and down across 400 μm near the well floor (Fig. 2A). In this way, any gradient in O_2_ concentration correlating to a cell density gradient driven by cell settling will be quantified. One full cycle of vertical scanning takes 15 min.

For each assay, the inoculating culture of *P. melaninogenica* was initially grown in Brain Heart Infusion (BHI) broth at 37°C under strictly anaerobic conditions (0% O_2_). One-hundred microliters of BHI inoculums derived from this culture and 100 μl BHI-negative controls were loaded into the center wells of a Cornell round-bottom 96-well plate under strictly anaerobic conditions (0% O_2_). Outer wells were loaded with PBS to mitigate evaporation. The 96-well plate was then transferred to the hypoxic chamber via an anaerobic transfer container. We let the plate sit for 1 h at 37°C to allow for cell settling. This step is important both to accurately mimic the biofilms formed by *P. melaninogenica in vivo* and to ensure accurate readings with the Resipher probe. During this time, it is also important that the plate acclimate to temperature and to the set atmospheric oxygen (2%, 5%, or 8%). One hour prior to this incubation, the Resipher device and sensing lid were placed in the hypoxic chamber to acclimate for 2 h prior to being loaded onto the 96-well plate. After the lid and device were loaded onto the plate, the assay was run for 45 h.

### Sample preparation and RNA sequencing

The inoculating culture of *P. melaninogenica* was initially cultured in Brain Heart Infusion (BHI) broth at 37°C under strictly anaerobic conditions (0% O_2_). Five biological replicates (*n* = 5) were established in BHI broth with a standardized starting optical density (OD_600_) of 0.05. Two identical aliquots were made from each biological replicate. These two sets of five inoculums were cultured simultaneously at 37°C under either 2% O_2_ or 0% O_2_ conditions. Cells were harvested during the exponential phase, with an average sample OD of ~0.36. Cultures were stored according to the Qiagen RNAprotect protocol until further processing. Total RNA was isolated using the Qiagen RNeasy Mini Kit. Quality control analysis confirmed that all samples met library standards, with DV200 values between 71% and 93% and ≥100 ng total RNA. Total RNA library prep with ribonucleotide depletion was performed. Short-read sequencing was performed using the Aviti24 high-output kit. Libraries were sequenced to a depth of ~20 million reads per sample.

### RNA sequencing and analysis

Raw paired-end RNA-seq reads were processed using fastp (version 1.0.1) for adapter detection and quality trimming. The software automatically identified paired-end adapter sequences (--detect_adapter_for_pe) and performed 5′ and 3′ sliding-window trimming to remove low-quality bases (--cut_front, --cut_tail, --cut_window_size 4, and --cut_mean_quality 20). Poly-G homopolymers were trimmed (--trim_poly_g), and reads shorter than 50 bp post-trimming were discarded. A transcriptome index for *Prevotella melaninogenica* ATCC 25845 (assembly GCA_000144405) was constructed using Salmon (version 1.10.3) with a k-mer size of 31. Trimmed reads were quantified using Salmon. For each sample, paired fastp-processed reads were supplied to Salmon quantification with selective alignment–based validation (--validateMappings) and GC-bias correction (--gcBias). Estimated counts from Salmon were combined into a gene count matrix. Gene-level count matrices were imported into R and analyzed using DESeq2 (version 1.46.0). Low-count genes were filtered according to DESeq2 defaults, size factors were estimated using the median-ratio method, and dispersion parameters were fit using DESeq2’s standard workflow (23, 47, 48).

### Protein homology analysis

BLASTp analysis of each identified putative oxidative stress defense protein in *P. melaninogenica* against genomes of the 21 *Prevotella* species and 13 reclassified species commonly found in the gut and vagina was performed. Protein hits within each species with >60% amino acid coverage and <0.05 E value were considered homologs and included in our analysis. The degree of homology was determined via amino acid similarity scores. Homologs were depicted with a heatmap, where the amino acid similarity is represented by color (100% similarity = orange; 0% similarity = ivory). The bacteria were then grouped into a dendrogram based on the homology results.

## Data Availability

Sequences related to this submission are available under BioProject accession number PRJNA1400211.

## SUPPLEMENTAL MATERIAL

Fig. S1 jb.00142-26-s0001.pdf Heat-killed OCR.

Fig. S2 jb.00142-26-s0002.pdf STRING analysis.

Table S1 jb.00142-26-s0003.pdf L2FC of oxidative stress genes.

Table S2 jb.00142-26-s0004.pdf L2FC of DNA repair genes.

Table S3 jb.00142-26-s0005.pdf Mean transcript counts.

ASM does not own the copyrights to Supplemental Material that may be linked to, or accessed through, an article. The authors have granted ASM a non-exclusive, world-wide license to publish the Supplemental Material files. Please contact the corresponding author directly for reuse.
