# Supplementary material for: Aerotolerant capacity of the lung symbiont Prevotella melaninogenica
Source: J Bacteriol. 2026 Jul 14;208(8):e00142-26. doi: 10.1128/jb.00142-26 (PMC13492135; doi:10.1128/jb.00142-26)
Supplement: Supplementary file 1 [file jb.00142-26-s0001.pdf]

Supplemental 1

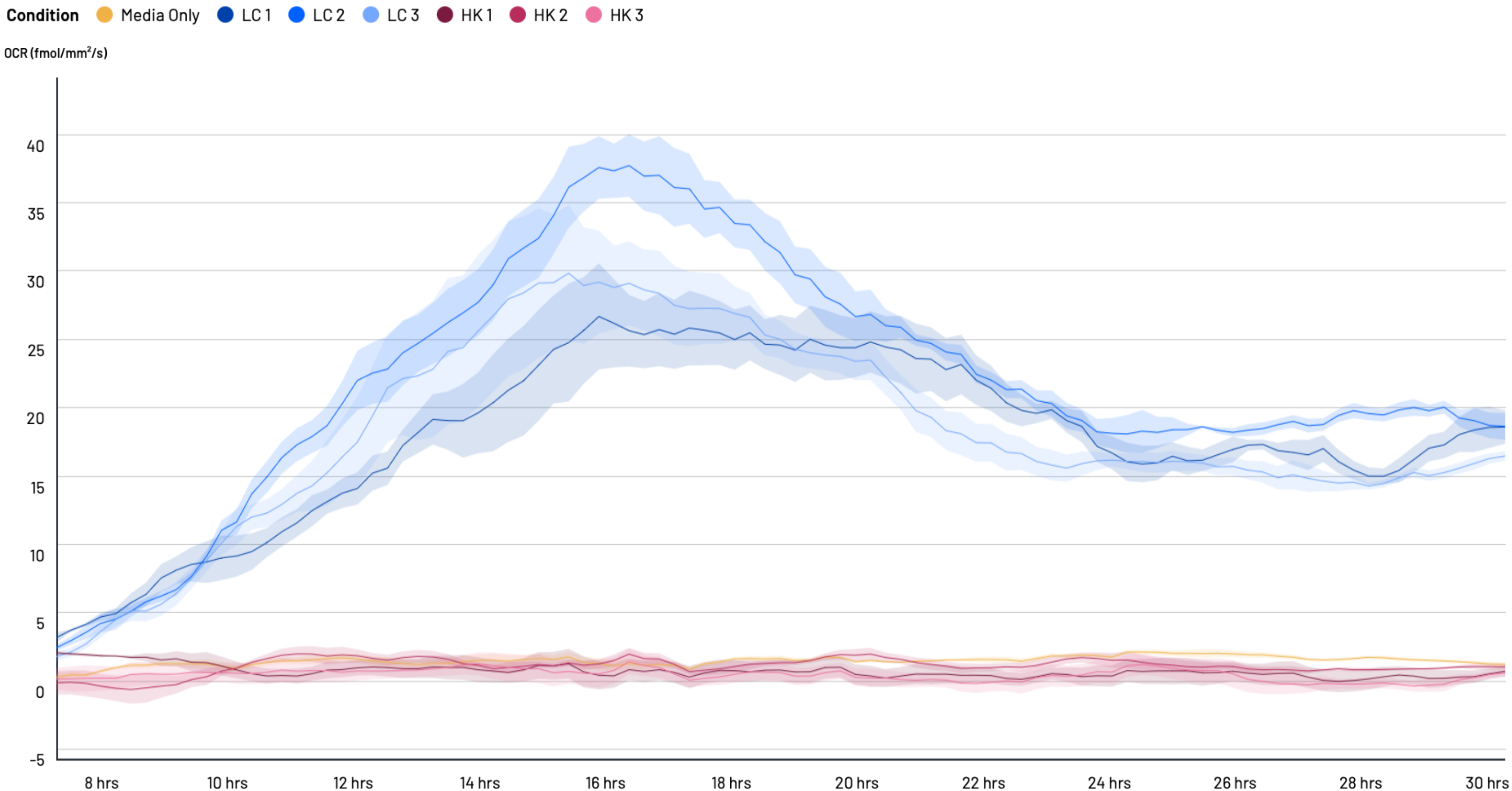

Sup 1. Heat-killed P. melaninogenica does not consume oxygen. The oxygen consumption rate (OCR) of heat-killed (HK) and live (LC) P. melaninogenica culture is shown under 5% atmospheric oxygen. Three biological replicates are shown for each condition, with error bars representing technical replicates. The heat killed condition is represented in red, live cells in blue, and the media only control in orange.
