# Supplementary material for: Aerotolerant capacity of the lung symbiont Prevotella melaninogenica
Source: J Bacteriol. 2026 Jul 14;208(8):e00142-26. doi: 10.1128/jb.00142-26 (PMC13492135; doi:10.1128/jb.00142-26)
Supplement: Supplementary file 2 [file jb.00142-26-s0002.pdf]

Supplemental 2

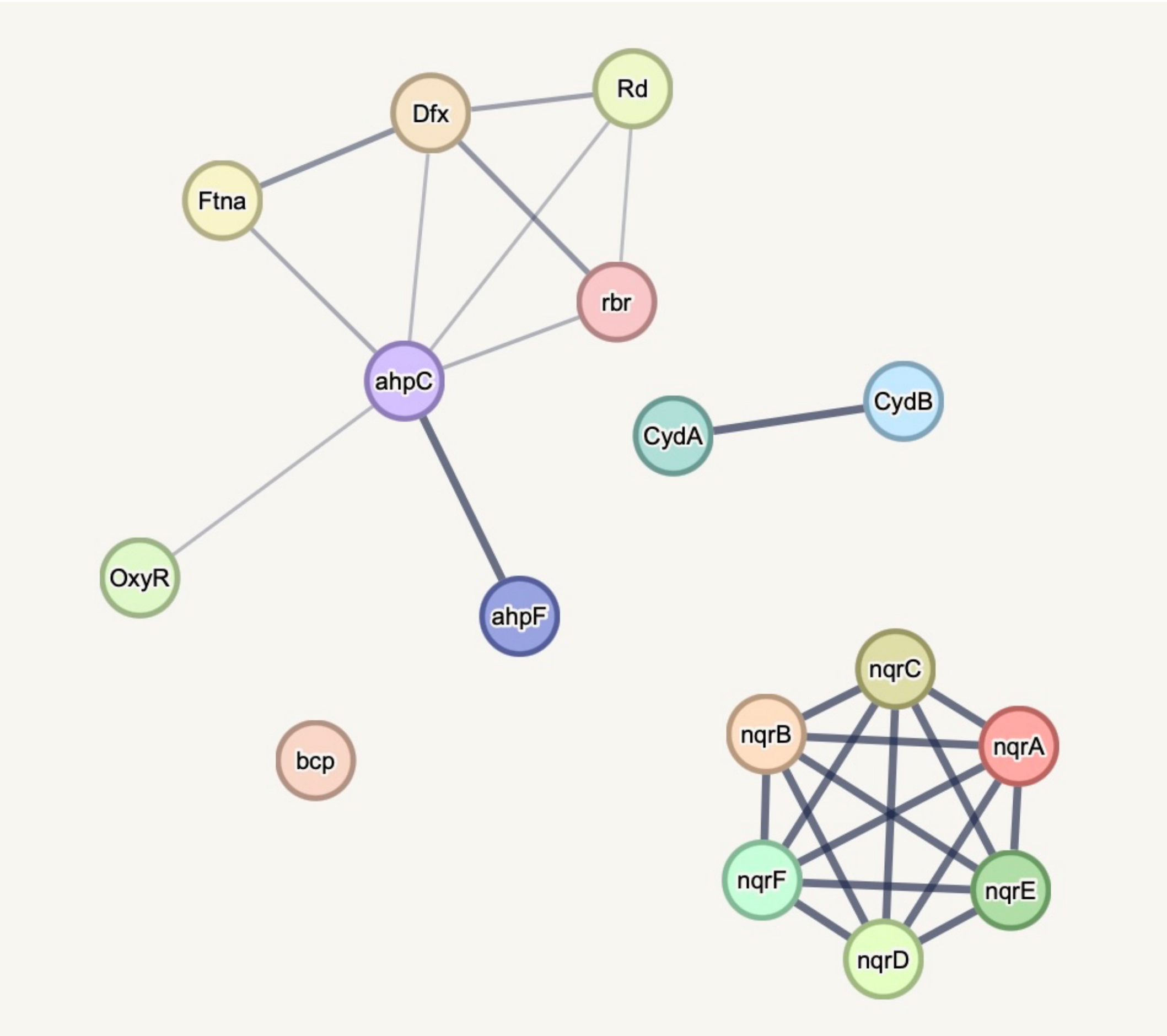

Sup 2. STRING analysis of protein interactions. Line thickness correlates to interaction confidence scores. Protein names are listed in each node. Database functions, genomic neighborhood, textmining, experimental evidence co-occurence co-expression, and gene fusion all determine confidence. Minimum required confidence for line = .400
