# Supplementary material for: Aerotolerant capacity of the lung symbiont Prevotella melaninogenica
Source: J Bacteriol. 2026 Jul 14;208(8):e00142-26. doi: 10.1128/jb.00142-26 (PMC13492135; doi:10.1128/jb.00142-26)
Supplement: Supplementary file 3 [file jb.00142-26-s0003.pdf]

Supplemental Table 1

Gene	log2FC
ahpF	4.2
ahpC	4.03
appC	1.7
cydB	1.59
rd	1.59
dfx	1.43
ftnA	1.04
bcp	0.97
nqrA	0.48
rbr	0.41
oxyR	0.35
nqrD	0.24
nqrC	0.21
nqrF	0.16
nqrB	0.16
nqrE	0.12

S 1. The positive log2 Fold Change of each putative oxidative stress defense gene under 2% oxygen.
