# Supplementary material for: Aerotolerant capacity of the lung symbiont Prevotella melaninogenica
Source: J Bacteriol. 2026 Jul 14;208(8):e00142-26. doi: 10.1128/jb.00142-26 (PMC13492135; doi:10.1128/jb.00142-26)
Supplement: Supplementary file 4 [file jb.00142-26-s0004.pdf]

Supplemental Table 2

Gene	log2FC
dps	*3.98*
recO	1.52
xerC_10	1.08
uvrA_1	0.71
ruvA	*0.7*
mutY	*0.67*
hupB	*0.48*
polA	*0.41*
ruvB_2	*0.37*
atl	*0.34*
dnaN	*0.33*
uvrA_2	*0.31*
radA	*0.3*
mfd	*0.3*
hup	*0.24*
uvrB	*0.14*
mutS2	*0.13*

S 2. The positive log2 Fold Change of each putative DNA repair gene under 2% oxygen.
