# Supplementary material for: Aerotolerant capacity of the lung symbiont Prevotella melaninogenica
Source: J Bacteriol. 2026 Jul 14;208(8):e00142-26. doi: 10.1128/jb.00142-26 (PMC13492135; doi:10.1128/jb.00142-26)
Supplement: Supplementary file 5 [file jb.00142-26-s0005.pdf]

Supplemental Table 3

Gene Symbol	Description	Transcript Count	Relative Abund.
rbr	Rubrerythrin	70994.1	
nqrA	Na(+)-translocating NADH-quinone reductase subunit A	22512.3	
nqrF	Na(+)-translocating NADH-quinone reductase subunit F	20539.0	
dfx	Desulfoferrodoxin (SOR)	18967.7	
nqrB	Na(+)-translocating NADH-quinone reductase subunit B	17606.8	
appC	Cytochrome bd-II ubiquinol oxidase subunit 1	15389.8	
ftnA	Bacterial non-heme ferritin	10008.3	
cydB	Cytochrome bd-I ubiquinol oxidase subunit 2	9976.5	
ahpC	Alkyl hydroperoxide reductase C -peroxidase	9952.7	
nqrE	Na(+)-translocating NADH-quinone reductase subunit E	8237.5	**8193 Average**
nqrC	Na(+)-translocating NADH-quinone reductase subunit C	7975.1	
nqrD	Na(+)-translocating NADH-quinone reductase subunit D	6839.3	
ahpF	NADH dehydrogenase	6534.1	
bcp	Peroxiredoxin Bcp	4275.8	**2815 Median**
oxyR	Hydrogen peroxide-inducible genes activator	2224.4	
rd	Rubredoxin	451.8	

S Table 3. The base mean transcript counts of target genes compared to the average and median transceript counts across all 2,307 predicted genes in P. melaninogenica. Gene symbols and descriptions are listed adjacent to their respective count and relative abundance compared to other genes. Almost all genes are above the average and median, with only one gene (rd) significanlty below the median.
